# Pediatric Mercury Intoxication Mimicking Pheochromocytoma

**DOI:** 10.4274/balkanmedj.2018.1025

**Published:** 2018-11-15

**Authors:** Marko Bjeloševič, Martina Fabianová, Peter Olejník, Pavol Kunovský

**Affiliations:** 1 Department of Pediatric Cardiology, Comenius University in Bratislava Faculty of Medicine, Bratislava, Slovakia; 2 Department of Arrhythmias and Cardiac Pacing, Pediatric Cardiac Center in Bratislava, Bratislava, Slovakia; 3 Department of Pediatrics, Comenius University in Bratislava Faculty of Medicine, Bratislava, Slovakia; 4 Intensive Care Unit of Pediatric Cardiology, Pediatric Cardiac Center in Bratislava, Bratislava, Slovakia

To the Editor,

A 10-month old boy with a low socioeconomic background presented with severe systemic hypertension (140/90 mmHg), tachycardia (160 beats/minute), profuse sweating, and cachexia at our cardiac intensive care unit on suspicion of pheochromocytoma. The patient had a 2-month history of failure to thrive, marked sweating, and motor regression. Other findings included depigmented patches of itchy skin (Figure 1a, 1b), palmar desquamation, weakness, hypotonia, and areflexia. The patient's history was otherwise insignificant. Continuous urapidil infusion was initiated in order to control the blood pressure.

Plasma metanephrine levels were normal-to-mildly elevated [metanephrine 0.30 (reference value: 0-0.46 nmol/L); normetanephrine 1.06 (0-0.98 nmol/L); epinephrine 0.73 (0-0.46 nmol/L); norepinephrine 4.45 (0-2.48 nmol/L); domapine <0.2 (0-0.55 nmol/L)]. NT-pro BNP was elevated [2083 ng/L (95^th^ percentile is 646 ng/L)]. Cortisol and thyroxine were minimally elevated, whereas renin, aldosterone and testosterone levels were normal. A CT scan did not reveal any abdominal tumors, nor renal artery stenosis. Electromyography showed almost no peripheral potentials. After excluding the possibility for Kawasaki disease and neoplasm, metabolic disease was considered.

Information presented regarding a 10-year old sibling being treated in a district hospital with similar cardiac symptoms was the impetus for considering a common etiology among both children. The combination of cardiac, neurological, and dermatological findings led us to suspect mercury poisoning; blood and urine levels were significantly elevated [blood 37 μg/L, urine 79 μg/L, and 500 μg/g creatinine (reference value: 0-0.8 μg/L, 0-0.4μg/L, 0-1 μg/g creat, respectively; conversion: 5 μg/L= 1 nmol/L)]. Additionally, elevated levels of cadmium [26 μg/L (reference value: 0-0.5 μg/L)] and traces of lead were observed. The patient's sister also positively tested for high levels of urinary Hg (137 μg/g creat). Both patients were started on intravenous chelation therapy with 2,3-dimercapto-1-propanesulfonic acid (Figure 1c). No adverse effects of this treatment were observed. The infant showed marked improvement with chelation, his blood pressure and heart rate normalized and he remains asymptomatic two years post-treatment. Asymptomatic family members were also tested, showing positive results in all of the other 5 siblings (4-14 years old, 34-256 μg Hg/g creat) and both parents (♂33 and ♀32 years old, 52 and 102 μg Hg/g creat respectively); all underwent oral chelation treatment. Written informed consent was obtained from the patient's parents.

Mercury (Hg) is an environmental pollutant and it is not involved in the physiological or biochemical processes of the body (1,2). Even with stricter policies, Mercury still poses a threat to public health, particularly among children, who are attracted by its appearance; demonstrated in Uysalol et al. (2,3,4). Hg use includes the manufacturing of batteries, fluorescent lights, semi conductors, paper, felt, and some paints. Frequent sources of current pediatric Hg intoxication include laboratory Hg (science laboratories within schools), household Hg (thermometers), alternative medicine (Ayurvedic/Chinese), and skin whitening creams (1,2,4,5,6).

Hg binding to co-enzyme S-adenosyl methionine results in inactivation of catechol-O-methyltransferase required for degradation of catecholamines (7). This leads to increased levels of catecholamine and catecholamine metabolites, which clinically mimics pheochromocytoma. Catecholamine levels are of diagnostic significance: the upper limit of normal values or only mildly elevated (two-times upper limit of normal) are typical for Hg intoxication, whereas at least four-times of upper limit of normal values are diagnostically typical of pheochromocytoma (1,2,4,6,8).

Hg interferes with DNA transcription and protein synthesis, resulting in palmar desquamation and a non-specific rash (4). Kawasaki disease is commonly considered; however, the clinical picture does not generally meet the full diagnostic criteria (8,9). Peripheral neuropathy, along with pain, tremor, itching, weakness, behavioral changes (developmental regression in infants), and convulsions are common. The first symptoms of chronic Hg intoxication are typically neurological and dermatological, and later, cardiovascular (1,2,4,6). The key to diagnosing rare diseases remains to carefully obtain a patient’s history: a sibling or a group of individuals presenting with similar symptoms is strongly suggestive of a common environmental etiology.

Our patient’s source of intoxication remains unknown - both water (tap and well) and air measurements were negative, and children from neighboring houses tested negative. With the given heavy metal combination (Hg, cadmium, lead), illegal battery recycling is the most likely etiology. Metals can be obtained by melting down batteries and the metals are sold for profit afterwards. A similar intoxication case was reported from household processing gold ore (10). We are concerned that illegal recycling practices among the economically disadvantaged are increasing, and that the medical consequences are potentially devastating. An early diagnosis of Hg poisoning is vital in helping to avoid unnecessary and invasive diagnostic tests, ensuring adequate treatment, and preventing long term damage.

In children presenting with unexplained cardiovascular findings (hypertension, tachycardia, etc.), rash and neurological symptoms, mercury intoxication mimicking pheochromocytoma should be considered in the differential diagnosis.

## Figures and Tables

**Figure 1:**
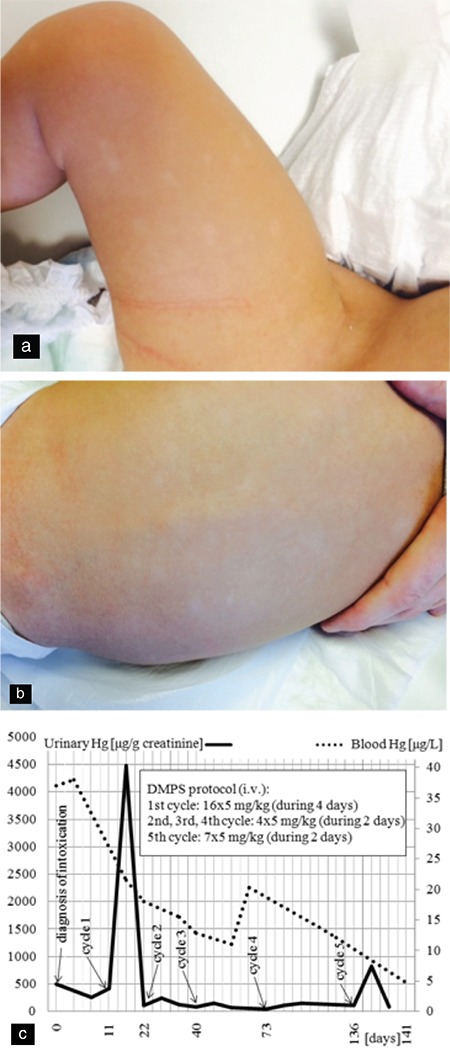
a-c. Depigmentations on the thigh (a), Depigmentations on the trunk and back (b), Blood and urinary mercury levels of the index patient during 5 DMPS cycles; Day 0= time of diagnosis of mercury intoxication (c). *DMPS: 2, 3-dimercapto-1-propanesulfonic acid*
